# Thyroid Hormone Induces PGC-1α during Dendritic Outgrowth in Mouse Cerebellar Purkinje Cells

**DOI:** 10.3389/fncel.2017.00133

**Published:** 2017-05-09

**Authors:** Tetsu Hatsukano, Junko Kurisu, Kansai Fukumitsu, Kazuto Fujishima, Mineko Kengaku

**Affiliations:** ^1^Kengaku Group, Institute for Integrated Cell-Material Sciences (WPI-iCeMS), Kyoto UniversityKyoto, Japan; ^2^Kengaku Group, Graduate School of Biostudies, Kyoto UniversityKyoto, Japan

**Keywords:** Purkinje cell, thyroid hormone, PGC-1α, mitochondria, dendritogenesis, hypothyroid

## Abstract

Thyroid hormone 3,3′,5-Triiodo-L-thyronine (T3) is essential for proper brain development. Perinatal loss of T3 causes severe growth defects in neurons and glia, including strong inhibition of dendrite formation in Purkinje cells in the cerebellar cortex. Here we show that T3 promotes dendritic outgrowth of Purkinje cells through induction of peroxisome proliferator-activated receptor gamma (PPARγ) co-activator 1α (PGC-1α), a master regulator of mitochondrial biogenesis. PGC-1α expression in Purkinje cells is upregulated during dendritic outgrowth in normal mice, while it is significantly retarded in hypothyroid mice or in cultures depleted of T3. In cultured Purkinje cells, PGC-1α knockdown or molecular perturbation of PGC-1α signaling inhibits enhanced dendritic outgrowth and mitochondrial generation and activation caused by T3 treatment. In contrast, PGC-1α overexpression promotes dendrite extension even in the absence of T3. PGC-1α knockdown also downregulates dendrite formation in Purkinje cells *in vivo*. Our findings suggest that the growth-promoting activity of T3 is partly mediated by PGC-1α signaling in developing Purkinje cells.

## Introduction

The thyroid hormone 3,3′,5-Triiodo-L-thyronine (T3) is a key regulator of growth and development. T3 acts primarily through nuclear thyroid hormone receptors (TRs) TRα1 or TRβ1/2, which mediate transcriptional control of target genes by binding to the thyroid hormone responsive elements (TRE) on DNA. Upon binding of T3, TRs undergo conformational change and release corepressors, while they recruit coactivator proteins which enhance chromatin remodeling for transcription activation (Wu and Koenig, 2000; Zhang and Lazar, 2000; Rosenfeld et al., 2006). The T3/TR signal controls mitochondrial biogenesis by regulating expression of the nuclear- and mitochondrial-encoded genes in various tissues. The target genes in the nuclear genome include peroxisome proliferator-activated receptor gamma (PPARγ) co-activator 1α (PGC-1α) which in turn induces cellular pathways for mitochondrial biogenesis and adaptive thermogenesis (Wu et al., 1999; Lehman et al., 2000; Lin et al., 2002; St-Pierre et al., 2003; Sinha et al., 2010). PGC-1α lacks DNA-binding activity but interacts with key transcription factors regulating metabolic genes, including nuclear respiratory factors (NRF-1 and NRF-2; Wu et al., 1999; Gleyzer et al., 2005), estrogen-related receptor-α (ERR-α; Huss et al., 2002; Schreiber et al., 2003), and PPARs (Vega et al., 2000). PGC-1α also binds to TRβ, implying its function as a coactivator as well as a downstream target of the T3/TR signaling (Puigserver et al., 1998; Wu et al., 2002).

In the developing brain, T3 is involved in the control of migration and process arborization of neurons, synapse formation, and differentiation of glial cells (Vincent et al., 1982–1983; Oppenheimer and Schwartz, 1997; Anderson, 2001). Congenital hypothyroidism thus leads to a syndrome termed cretinism in humans which is associated with permanent deficits in cognitive and sensorimotor functions. In rodents, differentiation of cerebellar neurons is most severely affected by perinatal hypothyroidism, among them a striking perturbation of the formation and maturation of Purkinje cell dendrites (Vincent et al., 1982–1983; Legrand, 1984). Consistently, T3 treatment promotes branching and synaptic differentiation of Purkinje cell dendrites *in vivo* and *in vitro* (Bernal, 2007; Koibuchi, 2008). It has recently been demonstrated that T3 directly affects dendritic development of Purkinje cells through activation of TRα1 (Heuer and Mason, 2003; Fauquier et al., 2014) and TRβ (Portella et al., 2010; Yu et al., 2015). However, the downstream target of T3/TR mediating dendritic development of Purkinje cells remains to be elucidated.

Purkinje cells develop highly branched dendritic arbors that receive tens of thousands of synaptic inputs in the cerebellar neural circuits. Each dendritic branch is fueled with mitochondria in order to maintain ATP-dependent ionic transport during synaptic and spontaneous activity. We have previously demonstrated that mitochondria are actively transported in emerging dendrites and regulate local actin dynamics necessary for dendritic outgrowth in developing Purkinje cells (Fukumitsu et al., 2015). Furthermore, mitochondrial fission regulated by dynamin-related GTPase Drp1 is necessary for supplying mitochondria in extending dendrites (Fukumitsu et al., 2016). In order to increase mitochondrial mass to fill the expanding dendritic volume, however, not only fission/fusion dynamics but mitochondrial biogenesis must be upregulated during dendrite formation.

In this study, we investigated the mechanistic link between the T3-induced mitochondrial biogenesis and dendritic development in Purkinje cells. We demonstrate that T3 enhances mitochondrial biogenesis and dendritic growth, in part, through induction of PGC-1α in Purkinje cells.

## Materials and Methods

### Reagents

Commercial sources for reagents used for supplemental experiments were as follows:

3,3′,5-Triiodo-L-thyronine (T3; Sigma-Aldrich), 2-Mercapto-1-methylimidazole (MMI; Sigma-Aldrich), Sodium perchlorate monohydrate (PM; Sigma-Aldrich).

### Mice

Pregnant ICR mice and pups of either sex (Nihon-SLC) were used in this study. Mother and pups were housed individually *ad libitum* under a 12:12 h light-dark cycle at 23°C. The induction of developmental hypothyroidism was performed as previously described (Sawant et al., 2015). Pregnant and nursing mother mice were treated with 0.08% MMI, 1.0% PM and 5.0% sucrose in drinking water from day 18 of gestation until postnatal day 14 (P14). A control group was treated with 5.0% sucrose in water. All experiments were handled in agreement with guidelines of the Animal Experiment Committee of Kyoto University.

### Plasmids

pAAV-CAG-EGFP, pAAV-CAG-tdTomato, and pAAV-CAG-Mito-EGFP were constructed as previously described (Kaneko et al., 2011; Fukumitsu et al., 2015). PGC-1α cDNA (GenBank: AF049330.1) was amplified from a mouse brain cDNA library using the following primers; 5′-ggatccGCCACCATGGCTTGGGACATGTGCAG and 5′-ctcgagCCTGCGCAAGCTTCTCT. PGC-1α mutant cDNA, which contains three silent mutations introduced in the respective short hairpin RNA (shRNA) target sequence, was generated using the QuikChange Site-Directed Mutagenesis Kit (Agilent Technologies). The wildtype and shRNA-resistant mutant cDNAs were fused with mCherry and inserted into the pAAV-CAG vector. PGC-1α shRNA target sequence (5′-GAAGATAGATGAAGAGAATGA) was designed with the Web-based software siDirect. The DNA oligonucleotides containing the shRNA target sequence, a seven nucleotide loop region (tgtgctt), and the shRNA antisense sequence were ligated and cloned into pAAV-hH1 modified to express EGFP under a CAG promoter. pAAV-hH1 expressing a scrambled shRNA (5′-GTAAAGGAAATAGAGAAGAGT) was used as a negative control. pAAV-EGFP-NRF1DN was created by insertion of a deletion mutant of NRF1 (1–304 aa; GenBank: AF098077.1; Wu et al., 1999) amplified from a mouse brain cDNA library into the pAAV-CAG-EGFP vector. The full length RIP140 cDNA (GenBank: NM_173440.2) was amplified from a mouse brain cDNA library and fused with FLAG-tag to create pAAV-FLAG-RIP140.

### Primary Culture, Adeno Associated Virus and Electroporation

Primary cultures of cerebellar neurons were performed as described previously with slight modification (Fujishima et al., 2012). P0 mouse cerebella were dissected and dissociated in DMEM/F12 supplemented with 10% fetal bovine serum and plated on glass-based culture dishes coated with poly-D-lysine. After 2 h, cell cultures were washed with DMEM/F12 to remove FBS, and media were replaced by serum-free maintenance medium (DMEM/F12 containing 1% penicillin–streptomycin, 3.9 mM glutamine, 2.1 mg/ml glucose, 0.1 mg/ml bovine serum albumin, 30 nM selenium dioxide, 20 μg/ml insulin, 40 nM progesterone, 100 μM putrescine, 200 μg/ml transferrin). Ten nanomolar of T3 was added at 0 and 5 days *in vitro* (DIV). Plasmids were transfected to dissociated cells by using Amaxa Mouse Neuron Nucleofector Kit. Mito-EGFP was introduced to Purkinje cells by infecting adeno-associated viruses (AAV) at 0 DIV. AAV (10^9^–10^10^ plaque-forming units) was purified by using AVB Sepharose High Performance.

### *In Utero* Electroporation

*In utero* electroporation for Purkinje cells was performed as described previously (Nishiyama et al., 2012; Fukumitsu et al., 2015). Briefly, pregnant mice on day 11.5 of gestation were anesthetized by an intra-abdominal injection of somnopentyl (Kyoritsu). The plasmid DNA solution (5 μg/μl) purified using the Qiagen Plasmid Maxi Kit was injected by using an aspirator tube (Drummond) into the fourth ventricle of embryos. The positive electrode (CUY650P3; Nepa Gene) was placed on the anterior end of the fourth ventricle through the uterus. Four current pulses (amplitude, 33 V; duration, 30 ms; intervals, 970 ms) were delivered with a square-wave electroporation generator (CUY21; Nepa Gene).

### Immunofluorescence

Detailed protocols for immunofluorescence were described in a previous report (Kaneko et al., 2011). Antibodies used for immunofluorescence were as follows: mouse anti-Calbindin D28K (Swant): rabbit anti-Calbindin D28K (Millipore): goat anti-Calbindin (Santa Cruz): mouse anti-Pyruvate dehydrogenase (PDH; Abcam): rabbit anti- PGC-1α (Abcam): rabbit anti-DsRed (Clontech): mouse anti-Pax-6 (R&D): rabbit anti-cytochrome C oxidase IV (COX-IV; Abcam); and Alexa 488-, Alexa 568-, or Alexa 647-conjugated anti-mouse, anti-rabbit, and anti-goat IgG (Invitrogen).

### Image Acquisition and Analysis

Immunofluorescence images were taken with a confocal microscope (FV1000; Olympus) with a 40×, 60× or 100× objective (NA 0.95, 1.20 or 1.40, respectively). For morphometric analysis, dendrites were traced with Neurolucida software (MBF Bioscience). The dendritic area was quantified with ImageJ. For quantification of dendrite mitochondrial content (ratio of mitochondrial area to dendrite area), acquired images were thresholded, and area occupied by Mito-EGFP signal was divided by the entire dendritic volume by using ImageJ. PDH signal was calculated as the sum of PDH absolute value in cells labeled with volume markers. PGC-1α signal in the cell soma was averaged in each cell. The length and number of dendritic protrusions were measured in the dendritic segment of 10 μm in length from the distal tips using ImageJ software.

### Statistical Analysis

Data were analyzed using Student’s *t* test for single comparisons and by one-way or two-way analysis of variance (ANOVA) with Tukey’s HSD *post hoc* or Tukey-Kramer HSD analysis for multiple comparisons.

## Results

### T3 Induces Dendritic Development and Mitochondrial Biogenesis in Cultured Purkinje Cells

**Figure 1:**
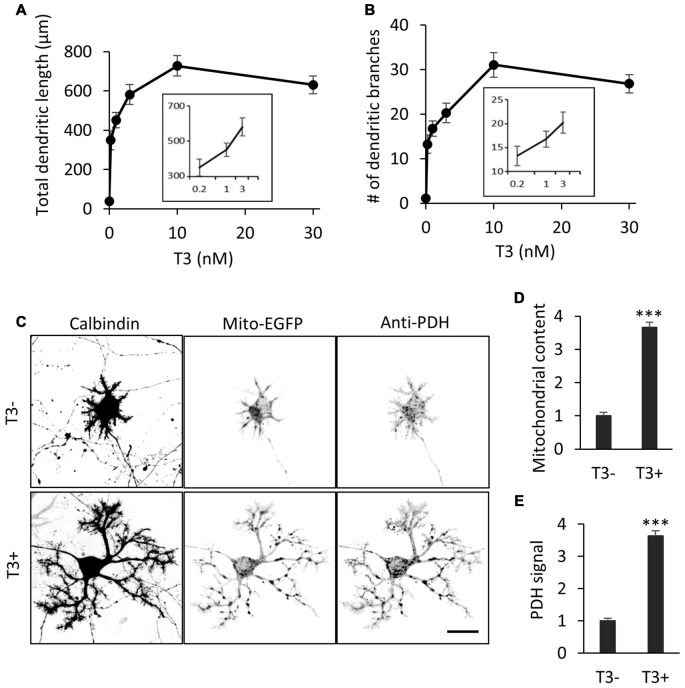
**3,3′,5-Triiodo-L-thyronine (T3) enhances mitochondrial biogenesis and dendritic outgrowth in cerebellar Purkinje cells.** Primary cerebellar cell cultures prepared from P0 mice were incubated for 10 days in the absence or presence of different concentrations of T3. The morphology of Purkinje cells was visualized by immunostaining with anti-Calbindin. **(A,B)** Dose-dependent effects of T3 on total length **(A)** and branch number **(B)** of Purkinje cell dendrites at 10 DIV. **(C)** Purkinje cells were labeled with adeno-associated viruses (AAV)-Mito-EGFP to visualize mitochondria and cultured with or without 10 nM T3. Cells were costained with anti-Calbindin and anti-pyruvate dehydrogenase (PDH) antibodies. Scale bar, 20 μm. **(D,E)** Quantification of mitochondrial content **(D)** and PDH expression **(E)** in Purkinje cells with or without T3 treatment. Signal intensity was normalized to the value of cells in the T3-deficient (T3-) condition. *N* = 30 for all data points. Data represent mean ± SEM; ***p < 0.001, Student’s *t* test.

We first confirmed the effect of T3 on dendritic development in cultured Purkinje cells. Cerebellar cells from P0 mice were dissociated and cultured in a serum-free medium with or without addition of T3 at different concentrations. The cultures were fixed at day 10 *in vitro* (10 DIV) and stained with an anti-Calbindin antibody for the morphometry of Purkinje cell dendrites. Consistent with previous studies (Heuer and Mason, 2003; Boukhtouche et al., 2010), T3 increased the total length and branch number of Purkinje cell dendrites in a dose-dependent manner, reaching a peak at 10 nM (Figures 1A,B).

To determine if T3-induced dendritic outgrowth is accompanied by an increase in dendritic mitochondria, we expressed the mitochondrial marker Mito-EGFP in Purkinje cells in culture using an adeno-associated virus vector-2 (AAV-2). The majority of mitochondria were located in the soma, while some were delivered to thin dendritic processes, in the cells cultured in the absence of T3. In contrast, mitochondrial mass was significantly increased in the cells treated with 10 nM T3, with enhanced delivery in extended dendritic branches (Figures 1C,D). T3 treatment also increased the expression of the mitochondrial enzyme PDH in dendrites as well as the soma (Figures 1C,E). Thus, T3-induced dendritic outgrowth is associated with increased generation and activity of mitochondria.

### T3 Induces PGC-1α Expression in Purkinje Cells during Dendritic Development

**Figure 2:**
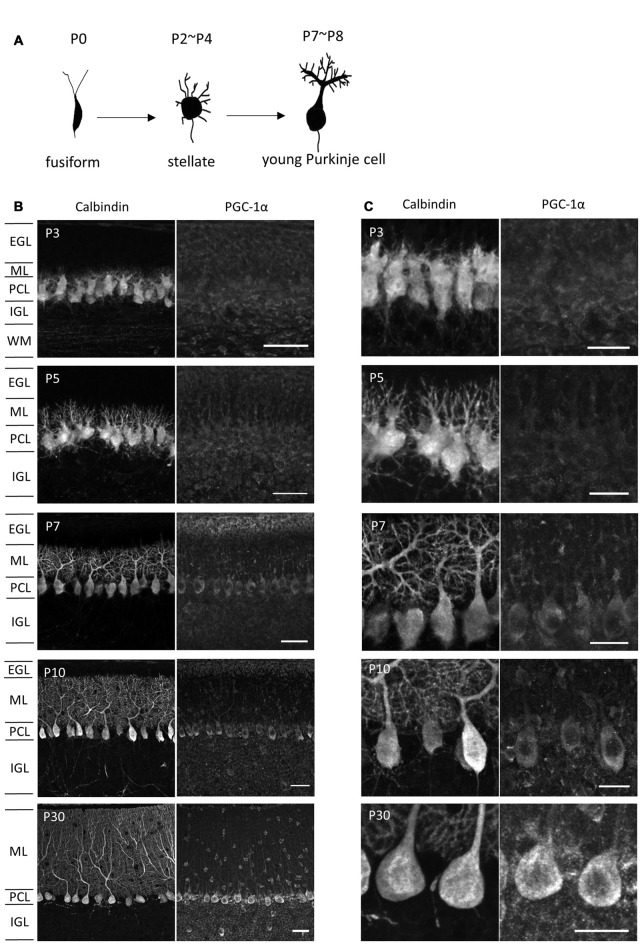
**PGC-1α expression in the developing cerebellar cortex. (A)** Shape changes of Purkinje cell dendrites during postnatal development. **(B,C)** Sagittal cerebellar sections were immunostained for PGC-1α and Calbindin at different ages of development and were observed at low **(B)** and high **(C)** magnification. Scale bars, 40 μm **(B)** and 20 μm **(C)**. PGC-1α was predominantly detected in the Purkinje cells from P7. EGL, external granule layer; ML, molecular layer; PCL, Purkinje cell layer; IGL, internal granule layer; WM, white matter.

It has been shown that PGC-1α regulates mitochondrial density in neurons (Wareski et al., 2009; Cheng et al., 2012; Vaarmann et al., 2016). To assess the involvement of PGC-1α in the increased mitochondrial generation and activity during dendritic development, we monitored PGC-1α expression in Purkinje cells of various developmental stages. Developing Purkinje cells undergo multiple steps of cell shape remodeling (Armengol and Sotelo, 1991; Boukhtouche et al., 2006; Takeo et al., 2015). In mice, Purkinje cells entered the Purkinje cell layer as elongated fusiform cells in prenatal stages. The first remodeling at P2-P4 is the retraction of the thin apical processes and emergence of numerous immature dendrites to become “stellate cells”. At the second remodeling at P7-P8, these immature processes are regressed and instead a thick primary dendrite with intricate branches emerges in young Purkinje cells (Figure 2A). As shown in Figures 2B,C, immunofluorescence revealed very weak expression of PGC-1α in the cerebellar cortex until postnatal day 5 (P5). PGC-1α expression was upregulated in Purkinje cells during the second remodeling around P7 when rapid extension of a single primary dendrite is initiated. After P10, PGC-1α was kept high in mature Purkinje cells as reported previously (Cowell et al., 2007). PGC-1β, a close family member which may function redundantly with PGC-1α, was not detected in Purkinje cells at the stages examined (data not shown).

**Figure 3:**
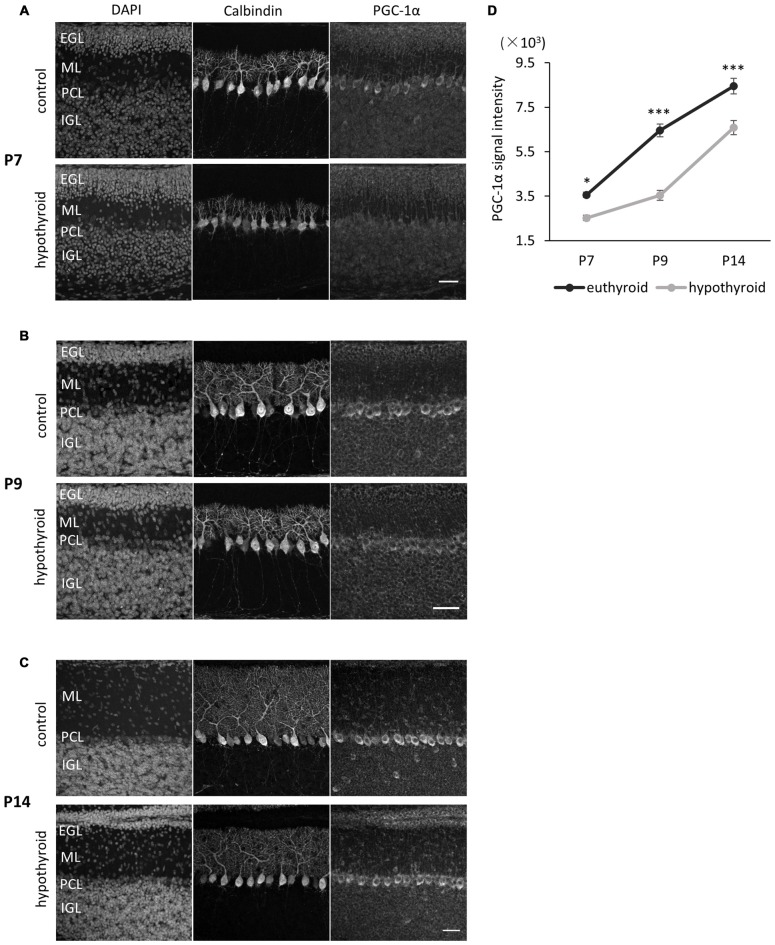
**PGC-1α expression in Purkinje cells is downregulated in hypothyroid mice. (A–C)** PGC-1α expression was compared at P7 **(A)**, P9 **(B)** and P14 **(C)**. Representative images from four mice in each condition are shown. Dendritic growth of Purkinje cells is retarded in the hypothyroid condition. At P14 **(C)**, the EGL is thicker in hypothyroid animals compared to control animals. Each section was immunostained with anti-Calbindin and anti-PGC-1α. DNA was counterstained with 4′,6-diamidino-2-phenylindole (DAPI). Scale bars, 40 μm. **(D)** Quantitative comparison of PGC-1α expression in Purkinje cells in control (euthyroid) and hypothyroid animals. Data represent mean ± SEM, *N* = 15 cells from four mice from two independent experiments for each points, *p < 0.05, ***p < 0.001, two-way analysis of variances (ANOVA) with Tukey’s HSD *post hoc* analysis.

To explore if the PGC-1α expression in Purkinje cells is regulated by ambient thyroid hormone in the developing cerebellum, we measured PGC-1α expression in Purkinje cells in hypothyroid mice. Developmental hypothyroidism was induced by treatment with antithyroid substrate MMI and sodium PM through the drinking water of pregnant dams from gestation day 18 through to lactation. The model mice exhibited mild hypothyroid phenotypes with decreased body size and prolonged presence of the EGL and dendritic growth defects in Purkinje cells as previously reported (Nicholson and Altman, 1972a,b; Morte et al., 2002; Figures 3A–C). Concomitantly, the expression of PGC-1α in Purkinje cells was significantly downregulated in the hypothyroid mice during postnatal development. In normal euthyroid mice, PGC-1α expression constantly increased from P7 to P14 (Figure 3D). In contrast, PGC-1α levels remained low until P9 in hypothyiroid Purkinje cells. PGC-1α expression was increased but still significantly lower than euthyroid mice at P14 (Figure 3D). These results suggest that T3 induces the onset of PGC-1α expression in Purkinje cells around P8, while other factors may regulate PGC-1α expression in later stages of cerebellar development.

**Figure 4:**
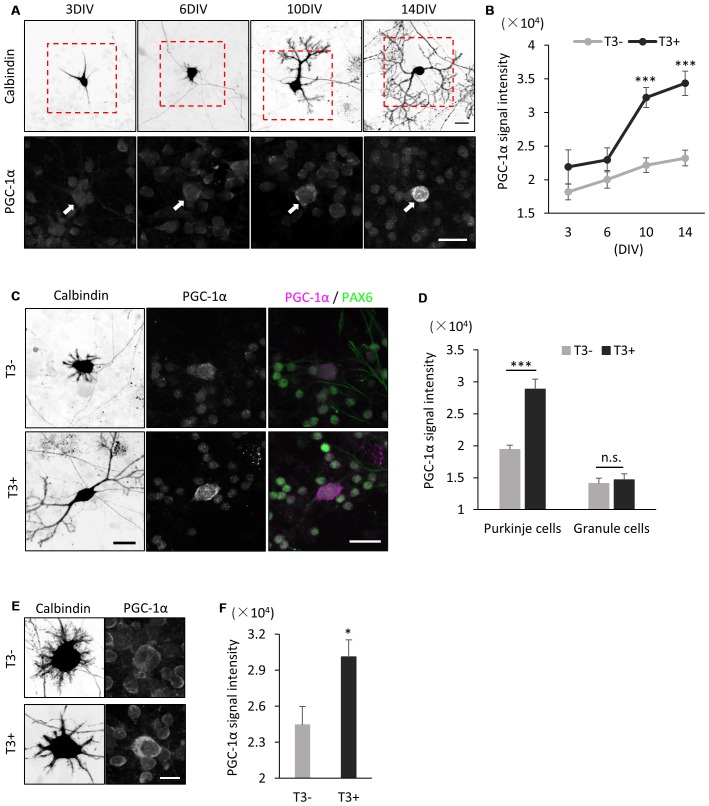
**PGC-1α expression is induced in cultured Purkinje cells by T3 treatment. (A)** Cerebellar cells were cultured in the presence of 10 nM T3 and then immunostained with anti-Calbindin and PGC-1α at the indicated day in culture. Boxed regions in the upper panels are enlarged in lower panels. PGC-1α expression is gradually increased in Purkinje cells (arrows) from 6 DIV. Scale bars, 20 μm. **(B)** Quantitative comparison of PGC-1α expression in Purkinje cells cultured with (black dots and line) or without (gray dots and line) T3. Data represent mean ± SEM, *N* = 15 cells for each points, ***p < 0.001, two-way ANOVA with Tukey’s HSD *post hoc* analysis. **(C)** Dissociated cerebellar cells were cultured with or without T3 treatment and immunostained for Calbindin, Pax-6 and PGC-1α at 10 DIV. Scale bars, 20 μm. **(D)** Quantitative comparison of PGC-1α expression in Purkinje cells and granule cells cultured with or without T3. **(E,F)** Immunofluorescent images **(E)** and quantitative comparison **(F)** of PGC-1α expression in Purkinje cells treated with or without T3 for 24 h. Scale bar, 10 μm. **(D,F)** The average pixel intensities of PGC-1α signals in the cell soma were measured. Mean ± SEM, *N* = 15 for each point, *p < 0.05, ***p < 0.001, Student’s *t* test.

We further analyzed the effect of T3 on PGC-1α expression in Purkinje cells in dissociated culture. In cultures treated with T3 (10 nM), PGC-1α signals were upregulated from 6 DIV and sharply increased until 14 DIV (Figures 4A,B). In contrast, PGC-1α signals were kept low in the culture without T3 treatment with slight increase during Purkinje cell development from 3 DIV to 14 DIV (Figure 4B). Furthermore, upregulation of PGC-1α levels by T3 was specific for Purkinje cells, while little or no change was detected in granule cells in the culture with or without T3 (Figures 4C,D).

To further confirm that PGC-1α expression is induced by T3, we analyzed if PGC-1α expression is upregulated in Purkinje cells immediately after exposure to T3. To this end, we cultured cerebellar cells in the absence of T3 and treated them with T3 at 9 DIV. We detected significant increase in PGC-1α expression in Purkinje cells at 24 h after T3 treatment (Figures 4E,F). Taken together, these results strongly suggest that T3 regulates the onset of PGC-1α expression in developing Purkinje cells.

### PGC-1α Is Required for Dendritic Outgrowth Induced by T3

**Figure 5:**
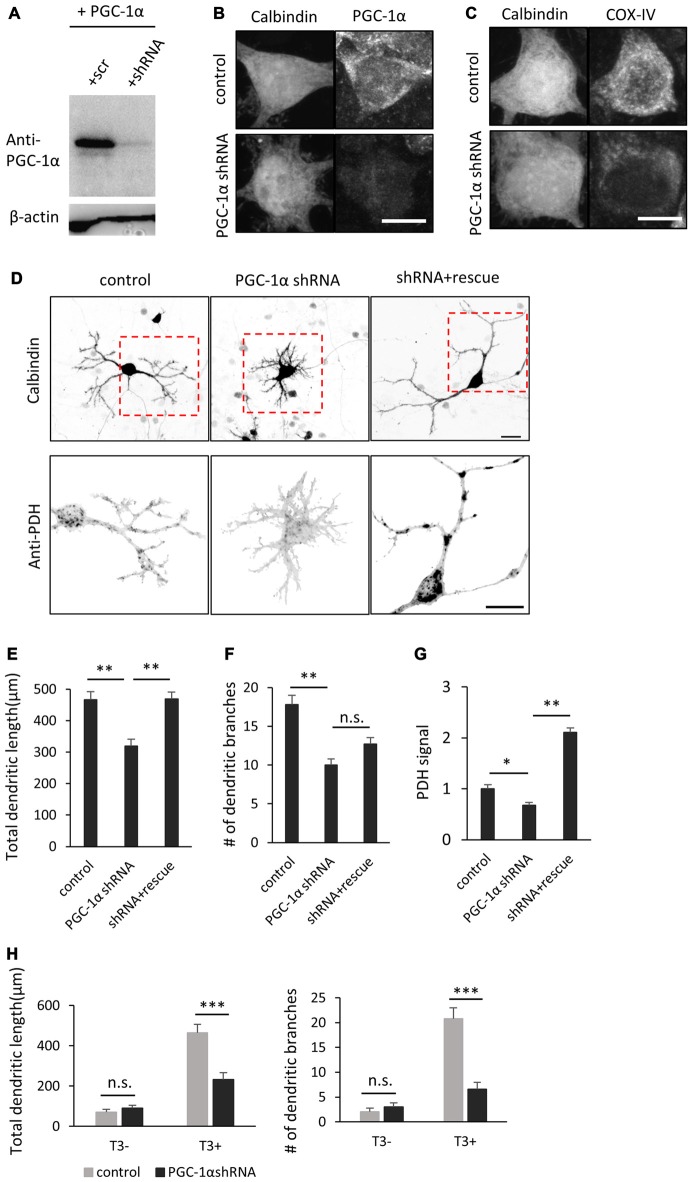
**PGC-1α knockdown inhibits T3-induced dendritic growth in Purkinje cells. (A)** HEK293T cells expressing mouse PGC-1α were transfected with PGC-1α-scramble (scr) or short hairpin RNA (shRNA) construct and analyzed by western blotting with anti-PGC-1α and anti-β-actin antibodies. **(B,C)** Cultured Purkinje cells were transfected with PGC-1α-shRNA or scr-shRNA (control) constructs and stained for Calbindin and PGC-1α **(B)** or Calbindin and cytochrome C oxidase IV (COX-IV) **(C)** at 10 DIV. Scale bars, 10 μm. **(D)** Representative images of Purkinje cells transfected with scr shRNA (control), PGC-1α shRNA (PGC-1α shRNA), or PGC-1α shRNA plus an shRNA-resistant mutant of PGC-1α (shRNA + rescue). Cells were cultured in the presence of 10 nM T3 until 10 DIV and immunostained with anti-Calbindin and anti-PDH antibodies. Boxed regions in the upper panels are enlarged in lower panels. Scale bars, 20 μm. **(E–G)** Quantitative analyses of the total dendritic length **(E)**, number of dendritic branches **(F)** and PDH signal **(G)**. Data represent mean ± SEM, *N* = 30 for each point, **p < 0.01 and *p < 0.05, one-way ANOVA with Tukey’s HSD *post hoc* analysis. **(H)** Quantification of the effects of PGC-1α knockdown on Purkinje cells morphology in the presence or absence of T3. Mean ± SEM, *N* = 15 for each point, ***p < 0.001, Student’s *t* test.

The above results indicate that T3 leads to increase in PGC-1α expression in Purkinje cells. To determine if PGC-1α activity is involved in T3-induced dendritic outgrowth, we performed knockdown of PGC-1α and examined its effect on dendrite morphogenesis in Purkinje cells. To do this, we first evaluated the efficacy of a shRNA in reducing the expression of exogenous PGC-1α transfected in HEK293 cells (Figure 5A). We then confirmed that the shRNA sequence efficiently suppressed endogenous expression of PGC-1α in cultured Purkinje cells (Figure 5B). PGC-1α shRNA also downregulated expression of COX-IV, a downstream target gene of NRF-1 which is activated in the presence of PGC-1α (Figure 5C). Specifically, Purkinje cells transfected with PGC-1α shRNA showed a marked decrease in the total length and branch number of dendrites in the presence of T3 (Figures 5D–F). PGC-1α knockdown also downregulated PDH signals in Purkinje cells (Figures 5D,G). In contrast, concomitant expression of shRNA with a shRNA-resistant mutant of PGC-1α recovered the dendritic length and PDH signals downregulated by the shRNA, though it did not fully rescue dendritic branching. Notably, PGC-1α knockdown inhibited dendritic outgrowth induced by T3, while it did not affect dendrites of untreated cells (Figure 5H).

**Figure 6:**
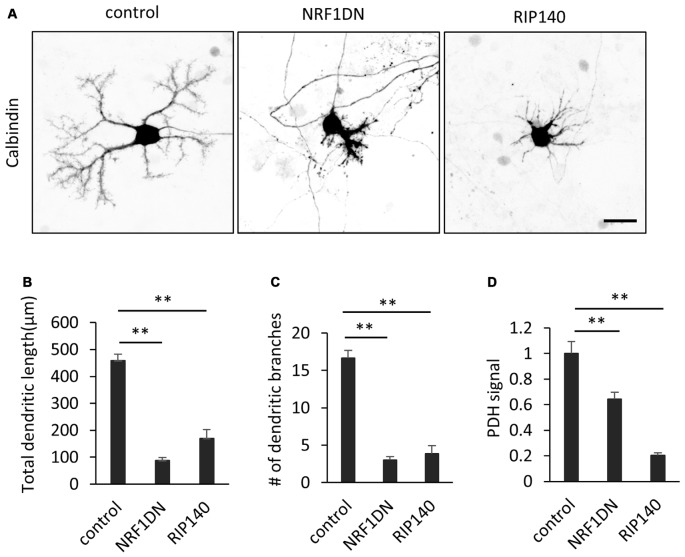
**Molecular perturbation of PGC-1α inhibits dendritic outgrowth and mitochondrial activity in Purkinje cells. (A)** Representative images of Purkinje cells overexpressing EGFP (control), EGFP-NRF1DN or FLAG-RIP140. Cells were stained for Calbindin at 10 DIV. Scale bar, 20 μm. **(B–D)** Quantitative analyses of the total dendritic length **(B)**, number of dendritic branches **(C)** and PDH signal **(D)**. *N =* 40 cells for control, 30 cells for NRF1DN and 30 cells for RIP140. Data represent mean ± SEM, **p < 0.01, one-way ANOVA followed by Tukey Kramer HSD tests.

We further confirmed the involvement of PGC-1α in dendritic outgrowth by molecular perturbation of PGC-1α signaling. NRF-1 is a major transcription factor which is bound and coactivated by PGC-1α in various cell types (Wu et al., 1999; Gleyzer et al., 2005). A deletion mutant of NRF-1 lacking the C-terminal transactivation domain has been shown to sequester PGC-1α and inhibit transcriptional activation of its downstream target genes (Wu et al., 1999). Indeed, Purkinje cells expressing the NRF-1 mutant (NRF1DN) exhibited hypoplastic dendrites and low PDH signals (Figures 6A–D). We also examined the effect of RIP140, a transcriptional corepressor of mitochondrial biogenesis, opposing PGC-1α function (Hock and Kralli, 2009). In addition, RIP140 and PGC-1α have been shown to competitively bind to TR (Wei and Hu, 2004). Consistently, overexpression of RIP140 significantly inhibited dendrite extension and PDH expression in Purkinje cells cultured in the presence of T3 (Figures 6A–D).

**Figure 7:**
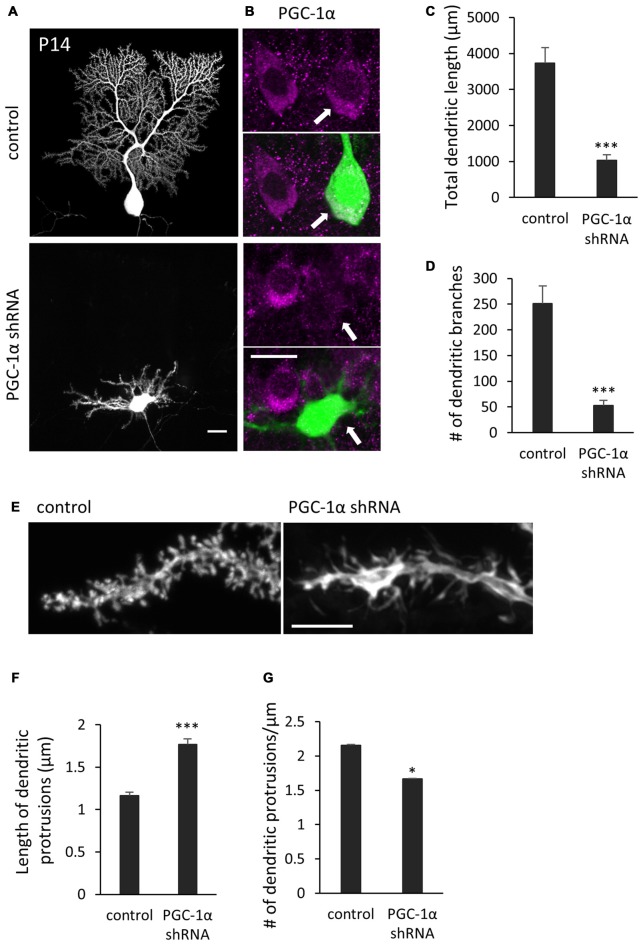
**Knockdown of PGC-1α inhibits dendritic outgrowth *in vivo* Purkinje cells. (A)** Representative images of Purkinje cells transfected with scr shRNA (control) or PGC-1α shRNA construct. Scale bar, 20 μm. **(B)** Dual color images of GFP derived from shRNA constructs (green) and immunostaining with anti-PGC-1α (magenta). Scale bar, 20 μm. **(C,D)** Quantitative analyses of the total dendritic length **(C)** and number of dendritic branches **(D)** in Purkinje cells expressing scr shRNA (control) or PGC-1α shRNA constructs. Data represent mean ± SEM, *N* = 12 cells from three mice, ***p < 0.001, Student’s *t* test. **(E)** Representative images of distal dendrites of Purkinje cells expressing scr shRNA (control) and PGC-1α shRNA. Scale bar, 5 μm. **(F,G)** The length **(F)** and number **(G)** of dendritic protrusions in the Purkinje cells expressing scr shRNA (control) or PGC-1α shRNA (shRNA). Data represent mean ± SEM, *N* = 200 (control) and *N* = 259 (shRNA). Twelve cells from three mice were analyzed for each group. Data represent mean ± SEM, ***p < 0.001, *p < 0.05, Student’s *t* test.

We next sought to determine if PGC-1α is required for dendritic outgrowth *in vivo*. To this end, we delivered the shRNA construct into the cerebellum of embryonic day 11.5-old mice, via electroporation *in utero*. Immunohistochemical analysis revealed that the PGC-1α shRNA significantly lowered, though not completely abolished, endogenous PGC-1α expression in Purkinje cells at P14 (Figure 7B). At this stage, normal Purkinje cells extend a single primary dendrite with elaborate branches which are covered with numerous spines. In contrast, Purkinje cells expressing PGC-1α shRNA had multiple shorter and less branched dendrites, many of which misoriented and failed to reach the pial surface (Figures 7A,C,D). Compared to stubby spines on normal cells, the stunted dendrites of PGC-1α-deprived Purkinje cells were covered with elongated, filopodia-like spines (Figures 7E–G).

These results together support that the cell-autonomous function of PGC-1α is required for the later steps of dendritic development in Purkinje cells in response to T3.

### PGC-1α Substitutes for T3 during Dendritic Outgrowth of Purkinje Cells

**Figure 8:**
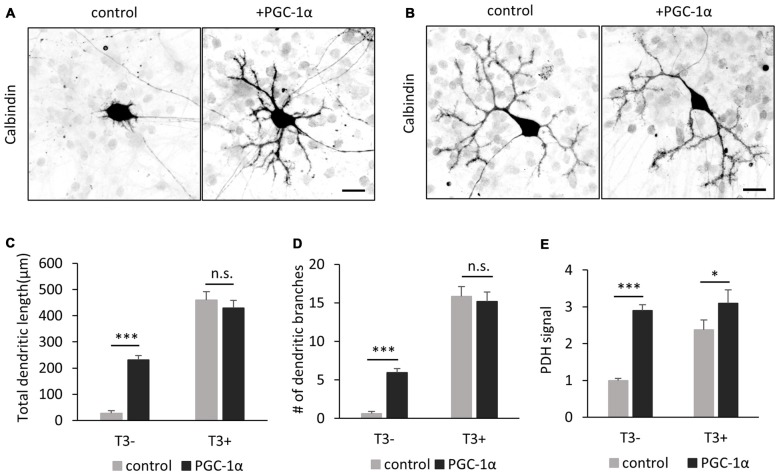
**PGC-1α overexpression enhances dendritic outgrowth of Purkinje cells in the absence of T3. (A,B)** The morphology of Purkinje cells transfected with tdTomato (control) or PGC-1α-mCherry (+PGC-1α). Cells were cultured with **(B)** or without **(A)** T3 and stained for Calbindin at 10 DIV. Scale bars, 20 μm. PGC-1α overexpression induced dendritic outgrowth in the absence of T3 **(A)**, but not in the presence of T3 **(B)**. **(C–E)** Quantitative analyses of total dendritic length **(C)** branch numbers **(D)** and PDH signal **(E)**. Purkinje cells expressing tdTomato (control) or PGC-1α were cultured with or without T3 treatment. *N* = 30 for all data points. Data represent mean ± SEM, ***p < 0.001 and *p < 0.05, Student’s *t* test.

To obtain further evidence for a link between T3-induced PGC-1α expression and dendrite extension, we next introduced PGC-1α into Purkinje cells cultured without T3. Overexpression of PGC-1α markedly rescued the defective dendrites and PDH expression in T3-depleted Purkinje cells (Figures 8A,C–E). On the other hand, exogenous expression of PGC-1α did not affect dendritic growth in control Purkinje cells supplemented with T3 (Figures 8B–E). These results substantiate the view that T3 promotes dendritic outgrowth in Purkinje cells at least in part by inducing PGC-1α signal.

## Discussion

Mitochondria produce the majority of cellular ATP via aerobic metabolism. The large expansion of growing dendrites in differentiating neurons should be accompanied by a rapid increase in mitochondrial mass and activity to handle their rising demand for energy. In the present study, we demonstrate that T3 enables intensive outgrowth of Purkinje cell dendrites partly by upregulating PGC-1α expression. We provide several lines of evidence that PGC-1α is a downstream target of T3 and enhances mitochondrial biogenesis and activity in the Purkinje cells treated with T3.

### T3 Induces Purkinje Cell Differentiation via Multiple Downstream Pathways

It has been reported that T3 action in Purkinje cells is mediated by retinoic acid receptor-related orphan receptor alpha (RORα; Boukhtouche et al., 2010). RORα is expressed in Purkinje cells from the embryonic stage until adulthood (Ino, 2004). RORα is involved in multiple steps of Purkinje cell differentiation, from the early steps of dendrite formation to the maturation and maintenance of spines (Takeo et al., 2015). Specifically, RORα is induced by T3 prior to dendritogenesis of Purkinje cells, and mediates the first cell shape remodeling from thin fusiform to stellate shape, which is completed by P4 in mice (Boukhtouche et al., 2006, 2010). On the other hand, PGC-1α expression begins around P7 when cells undergo the latter step of remodeling from stellate shaped cells to young Purkinje cells. Consistently, knockdown of PGC-1α does not affect Purkinje cell migration nor the first remodeling, but strongly inhibits dendrite extension and spine maturation after P7. It is therefore probable that RORα and PGC-1α mediate differential steps of dendrite formation and maturation downstream of T3.

### Transcriptional Activation of PGC-1α in Purkinje Cells

The PGC-1α promoter contains a far upstream TRE recognition site and thus could be a direct target of transcriptional activation by T3 receptors (Wulf et al., 2008). Consistently, depletion of T3 downregulates PGC-1α expression in Purkinje cells which begins around 6 DIV in culture and P7 *in vivo*. However, the causal relationship between the decrease in synapse formation in hypothyroid mice (Nicholson and Altman, 1972a,b) and the defects in spine maturation in PGC-1α knockdown cells is unclear, as PGC-1α expression is upregulated to some extent by P14 in the hypothyroid mice. It is likely that other factors regulate PGC-1α in the late stages of cerebellar development. Besides the TRE, PGC-1α promoter contains a cyclic AMP-responsive element (CRE) and MEF2 binding site, which are activated by Ca^2+^-induced signaling molecules. It has been reported that BDNF signaling activates the CRE site in the PGC-1α promoter during synaptogenesis in hippocampal neurons (Cheng et al., 2012). Other studies have implicated MEF2 in dendritogenesis and spine formation and function in various CNS neurons (reviewed by Brusco and Haas, 2015). As thyroid hormone regulates the production of BDNF (Neveu and Arenas, 1996; Koibuchi et al., 1999a) and other neurotrophins (Clos and Legrand, 1990; Lindholm et al., 1993; Neveu and Arenas, 1996), it is possible that T3 indirectly induces PGC-1α expression by enhancing neurotrophin production. Though we do not garner evidence for it here, these activity-dependent pathways may regulate PGC-1α expression during spine formation in later stages, whereas T3 may regulate spinogenesis independently of PGC-1α.

### Candidate Transcription Factors Coregulated by PGC-1α during Dendritogenesis of Purkinje Cells

PGC-1α interacts with multiple transcription factors to induce multiple classes of mitochondrial genes regulating mitochondrial biogenesis and oxidative metabolism. Among them, NRF-1/2 activates many mitochondrial genes, including nuclear mitochondrial genes regulating mtDNA replication, oxidative phosphorylation components, mitochondrial transporters and mitochondrial ribosomal proteins. We observe that the expression of a NRF-1 target gene, COX-IV, is downregulated in PGC-1α knockdown cells, suggesting that PGC-1α functions as a coactivator of NRF-1 in developing Purkinje cells (Figure 5C).

Besides regulating dendritic outgrowth by inducing mitochondrial biogenesis in Purkinje cells, PGC-1α might be involved in the second remodeling from stellate-shaped to monopolar cells, as revealed by the increase in the number of primary dendrites and perisomatic processes in PGC-1α knockdown cells (Figure 7A). These features are not observed in hypothyroid mice nor in cells deprived of dendritic mitochondria (Morte et al., 2002; Fukumitsu et al., 2015, 2016), but rather phenocopy the conditional deletion of RORα in Purkinje cells after P4 (Takeo et al., 2015). PGC-1α has been shown to act as a coactivator of RORα in liver and skeletal muscle cells (Liu et al., 2007). These observations raise a possibility that PGC-1α may induce genes downstream of RORα as its coactivator during the second remodeling, independently of mitochondrial biogenesis. Since RORα controls expression of various genes regulating Purkinje cell differentiation, including genes involved in calcium signaling and cytoskeletal function (Gold et al., 2003), PGC1α and RORα pathways might organize cell differentiation and metabolic state in a coordinated manner. Furthermore, RORα has been shown to bind to the TRE and may act as a transcriptional coactivator of T3/TRs downstream genes (Koibuchi et al., 1999b) in addition to its function as a downstream effector induced by T3 (Boukhtouche et al., 2010). T3, RORα and PGC1α thus likely form intricate cross-regulatory loop rather than simple signaling cascade. The mechanism by which PGC-1α enhances dendrite outgrowth remains to be fully elucidated.

## Author Contributions

KFk, KFj and MK conceived and designed the experiments. TH, JK and KFk performed the experiments. TH and JK analyzed the data. TH, KFj and MK wrote the manuscript. All authors read and approved the final manuscript.

## Funding

This work was supported by the KAKENHI (#26290005) of the Japan Society for the Promotion of Science (JSPS) and the Naito Foundation to MK.

## Conflict of Interest Statement

The authors declare that the research was conducted in the absence of any commercial or financial relationships that could be construed as a potential conflict of interest.
